# Frozen fresh blood plasma preserves the functionality of native human α_2_-macroglobulin

**DOI:** 10.1038/s41598-023-31800-8

**Published:** 2023-03-20

**Authors:** Soraia R. Mendes, F. Xavier Gomis-Rüth, Theodoros Goulas

**Affiliations:** 1 Proteolysis Lab, Molecular Biology Institute of Barcelona (CSIC), Barcelona Science Park, c/Baldiri Reixac 15-21, 08028 Barcelona, Catalonia Spain; 2 Department of Food Science and Nutrition, School of Agricultural Sciences, University of Thessaly, 43100 Karditsa, Greece

**Keywords:** Biochemistry, Biological techniques, Biophysics, Molecular biology

## Abstract

Human α_2_-macroglobulin (hα_2_M) is a large homotetrameric protein involved in the broad inhibition of endopeptidases. Following cleavage within a bait region, hα_2_M undergoes stepwise transitions from its native, expanded, highly flexible, active conformation to an induced, compact, triggered conformation. As a consequence, the peptidase is entrapped by an irreversible Venus flytrap mechanism. Given the importance of hα_2_M, biochemical studies galore over more than seven decades have attempted to ascertain its role, typically using authentic hα_2_M purified from frozen and non-frozen fresh blood plasma, and even outdated plasma. However, hα_2_M is sensitive once isolated and purified, and becomes heterogeneous during storage and/or freezing, raising concerns about the functional competence of frozen plasma-derived hα_2_M. We therefore used a combination of native and sodium dodecylsulfate polyacrylamide gel electrophoresis, affinity and ion-exchange chromatography, multi-angle laser light scattering after size-exclusion chromatography, free cysteine quantification, and peptidase inhibition assays with endopeptidases of two catalytic classes and three protein substrates, to characterize the biochemical and biophysical properties of hα_2_M purified ad hoc either from fresh plasma or frozen fresh plasma after thawing. We found no differences in the molecular or functional properties of the preparations, indicating that protective components in plasma maintain native hα_2_M in a functionally competent state despite freezing.

## Introduction

The α_2_-macroglobulin (α_2_M) family of large multi-domain proteins is found in animals and some colonizing bacteria^1–11^. The family is typified by human tetrameric α_2_M (hα_2_M), which is present at high concentrations in blood plasma. This protein appears to be essential because no total deficiency has been described, indicating that such a deficiency would be embryonically lethal^12–14^. The hα_2_M protein has many functions, including the transport of growth factors, cytokines, and hormones; the binding of misfolded and inactivated proteins as a chaperone; and the binding of metals^5,9,15–17^. In addition, the best-characterized function of hα_2_M is its broad-spectrum capacity to inhibit endopeptidases of the four major catalytic classes regardless of their specificity^13^. In this manner, hα_2_M regulates proteolysis in complex physiological contexts such as nutrition, haemostasis, signalling and tissue remodelling^2,8,10,11^, and mediates innate defence against external peptidases during envenomation and microbial infection^8,10,18–20^. Remarkably, the protein is an acute-phase protein in rodents^21^ but not in humans^22^.

Secreted hα_2_M is a glycosylated 1451-residue protein with 11 domains, which forms a ~ 675-kDa homotetramer comprising a dimer of disulfide-linked dimers. This large protein operates as a suicidal trap using a Venus flytrap mechanism, which is based on protomers adopting either an expanded or compact conformation^23^. In its native active state, hα_2_M is a large, highly flexible structure with the four protomers in the expanded conformation, forming wide openings of up to 70 × 50 Å that allow disparate plasma components to enter its large central lumen (~ 600 nm^3^). Peptidases that cleave within a flexible multi-target bait region within the hα_2_M bait region domain^24^ cause a massive conformational rearrangement of the tetramer, involving several intermediates with protomers in either the expanded or compact state. This eventually leads to an irreversibly induced or triggered state^25^, in which all four protomers are compact and the capacity to inhibit peptidases is lost^23^. This structure has a much smaller central lumen (~ 300 nm^3^) and features 12 narrow openings of up to 30 × 40 Å, through which the trapped prey peptidase cannot escape. Moreover, the native and triggered tetramers differ in size and shape^23,26,27^, which increases the mobility of the triggered form in native polyacrylamide gel electrophoresis (PAGE)^28^. Macromolecules that enter the native tetramer without cleaving the bait can leave by diffusion. Accordingly, hα_2_M functions as a molecular sieve, screening circulating proteins and selectively catching peptidases.

Once within hα_2_M, the trapped peptidase is still able to interact with small substrates and inhibitors that diffuse into the particle through the aforementioned narrow openings. Finally, the C-terminal receptor-binding domain of each protomer, which is cryptic in the native tetramer^23,27^, becomes exposed on the hα_2_M surface and is recognized by cell surface receptors such as the low-density lipoprotein receptor-related protein. This triggers receptor-mediated endocytosis and destruction of the hα_2_M–peptidase complex in the lysosomes^29^.

This sequence of events has been established by structural and biochemical studies over more than seven decades. The starting point of most of these studies was authentic hα_2_M isolated from human plasma because it is difficult to produce functional recombinant hα_2_M capable of peptidase inhibition^30–32^, with some notable exceptions^33–36^. Once isolated and purified, native hα_2_M tends to be instable in vitro^37–39^. These findings cast doubt on the suitability of thawed frozen fresh plasma (FR) as a source of native hα_2_M instead of non-frozen fresh plasma (NF), because the former might yield a heterogeneous, artefactual and damaged population of hα_2_M particles^40^. Here, we address this question by using a combination of biophysical and functional assays to compare the structure and functionality of hα_2_M prepared from FR or NF plasma.

## Results and discussion

### The overall conformation of native hα2M is unaltered by plasma freezing

The α_2_Ms belong to the wider thioester-containing protein family, which includes not only endopeptidase inhibitors but also complement proteins C3, C4 and C5; the cell surface antigen CD109; and thioester-containing proteins from animals^10,19,41^. A hallmark of these proteins is a C–X–E–Q motif (C^972^–Q^975^ in hα_2_M), which forms a relatively stable *β*-cysteinyl-*γ*-glutamyl thioester bond in the native state. Native hα_2_M protomer structures in the expanded conformation were determined by cryo-electron microscopy (cryo-EM), revealing that stability results from the location of the thioester bond in a hydrophobic cavity and its protection by the nearby receptor-binding domain in partially different conformations^23,27,42^.

Under physiological conditions, thioester hydrolysis occurs after the conformational rearrangement triggered by cleavage of the bait region, which exposes the thioester bond^23,26,27^. The latter may then be targeted by surface lysine residues of the trapped peptidase, which gives rise to an ε-(γ-glutamyl)-lysine that is covalently linked to hα_2_M, releasing a free cysteine (C^972^)^43,44^. However, this covalent entrapment is not essential for peptidase inhibition by hα_2_M^45,46^.

In the absence of proteolytic activation, high concentrations of small reactive nucleophiles such as hydroxylamine or methylamine (MA) can cleave the hidden thioester bond^43,44,47^. This causes a conformational rearrangement similar to (but slower to form than) the peptidase-induced structure, which is revealed by its greater mobility in native PAGE and distinct elution profile during size-exclusion chromatography (SEC). This reaction likewise gives rise to the free cysteine but the hα_2_M tetramer can no longer bind and inhibit peptidases, despite possessing an intact bait region^47^.

**Figure 1:**
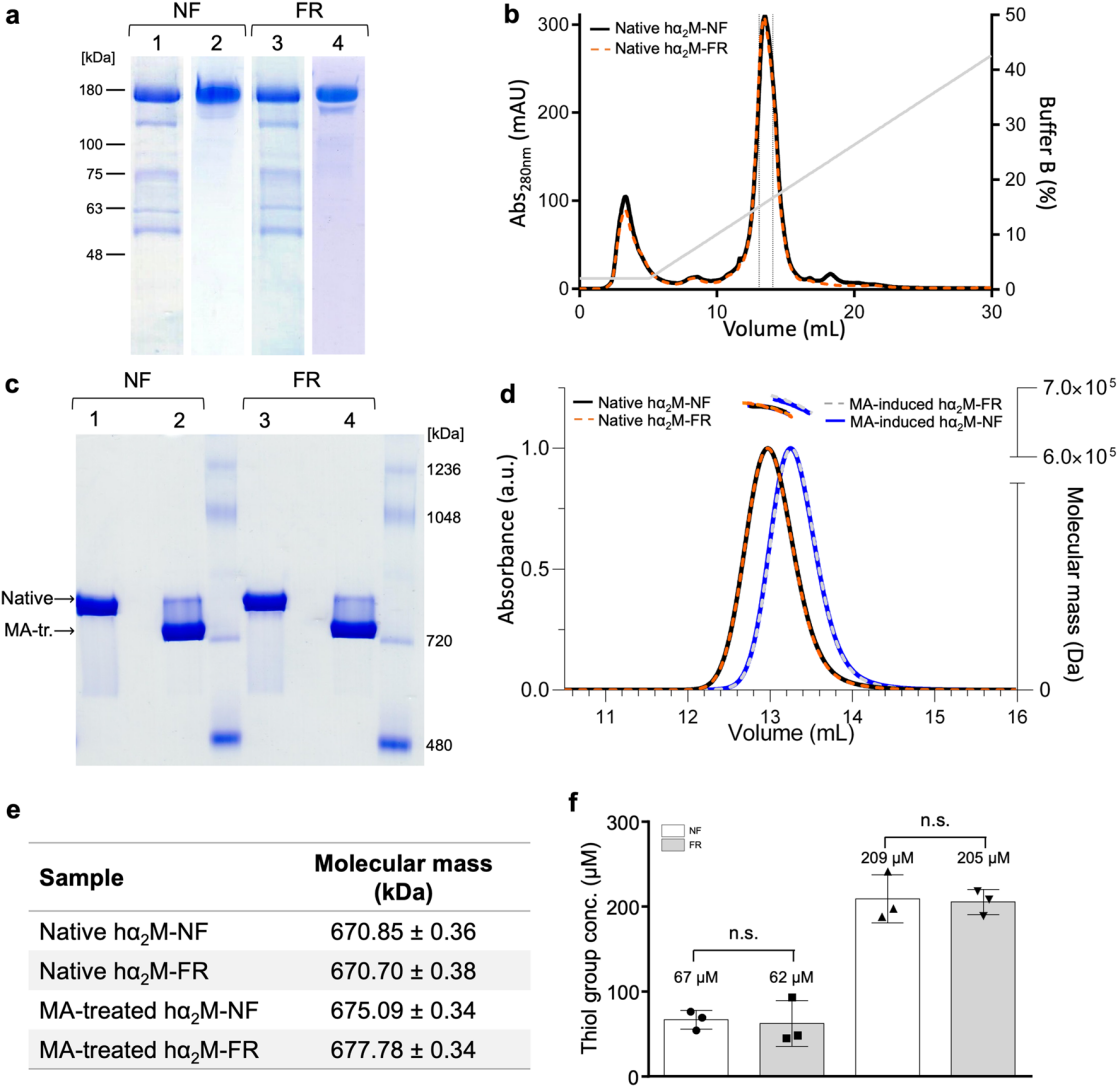
Purification and biophysical analysis of hα_2_M from non-frozen fresh plasma (NF) or frozen fresh plasma (FR). (**a**) SDS-PAGE analysis of elutes from zinc affinity chromatography (lanes 1 and 3) and IEC (lanes 2 and 4). (**b**) Comparison of the IEC profiles of native hα_2_M purified from NF (solid black line) and FR (dashed orange line). (**c**) Native PAGE analysis of native (lanes 1 and 3) and MA-treated induced (lanes 2 and 4) hα_2_M from NF (lanes 1 and 2) and FR plasma (lanes 3 and 4). (**d**) SEC–MALLS analysis of native (black and orange lines) and MA-treated induced (grey and blue lines) protein purified from FR (dashed lines) and NF (solid lines) plasma. (**e**) The molecular masses determined by SEC–MALLS were 671 kDa for the native tetrameric species and 675–678 kDa for the MA-treated species, which match the theoretical value (643.2 kDa) plus glycosylation. (**f**) Concentration of free thiol groups in hα_2_M from non-frozen (NF, white bars) or thawed frozen fresh (FR, grey bars) samples according to Ellmann’s reaction^51^. The protomeric hα_2_M concentration used was 157 μΜ. Data are presented as means ± SD (n = 3). Statistical significance was determined using a two-sided Student’s t-test (n.s., *p* > 0.1). Panels a-e are representative of more than three independent experimental replicates.

We compared the suitability of NF plasma and FR plasma, which is used for clinical transfusions as frozen fresh plasma preparations^48^, for the purification of hα_2_M using an established protocol based on polyethylene glycol (PEG) precipitation followed by zinc-affinity chromatography, ion-exchange chromatography (IEC) and SEC^23,31,49,50^. We analysed the zinc-affinity chromatography and IEC eluates by sodium dodecylsulfate PAGE (SDS-PAGE) (Fig. 1a) and compared the IEC profiles (Fig. 1b), revealing that the two hα_2_M preparations were equivalent. Next, we analysed the native and MA-treated samples by native PAGE and observed the aforementioned change in electrophoretic mobility, which was again indistinguishable between the two plasma sources (Fig. 1c). The MA-treated sample showed greater mobility than the native form in each case, as anticipated. Multi-angle laser light scattering after SEC (SEC–MALLS) showed the difference in migration between MA-treated and native samples, but the FR and NF preparations were again indistinguishable (Fig. 1d), also in terms of molecular mass before and after MA treatment (Fig. 1e). Overall, these results indicate that freezing blood plasma does not affect the conformation of hα_2_M, at least to the extent detectable using biophysical methods, and that hα_2_M from both sources appears well folded.

### Freezing blood plasma does not affect hα2M thioester bonds

To test the state of the thioester bonds in the native and MA-treated samples from FR and NF plasma, we quantified the free cysteines using Ellman’s reaction^51^ (Fig. 1f). The stoichiometry detected in the native preparations (62 μM and 67 μΜ) was equivalent and corresponded to a baseline reaction with cysteines other than C^972^, putatively from plasma molecules bound by hα_2_M (see Conclusions). In contrast, the MA-treated samples revealed an abrupt increase in the free thiol contents (205 μM and 209 μΜ). These concentrations corresponded to > 90% of the protomer concentration once the baseline was subtracted (i.e., close to one thiol group per protomer), which indicates nearly complete cleavage of the thioester bond by MA and the subsequent liberation of the C^972^ Sγ-atoms^44^. The values from the FR and NR samples were equivalent within experimental error.

### Plasma freezing does not impair the ability of native hα2M to inhibit peptidases

The Venus flytrap mechanism of inhibition is initiated by cleavage within the promiscuous bait region, which is exposed in the central lumen of native hα_2_M^23,26,52,53^. The efficiency of inhibition of hα_2_M is very high, so there is often a stoichiometric relationship between the number of bait regions cleaved and the molecules of peptidase inhibited. The limit is determined by the cavity size of the inner lumen of the induced tetramer, which can accommodate up to two peptidase molecules the size of trypsin^23,26^, and the overall shape of the peptidases. Sequestered peptidases remain able to cleave substrates small enough to access the lumen of the compact hα_2_M (and thus the active site of the bound peptidase) through any of the narrow openings.

**Figure 2:**
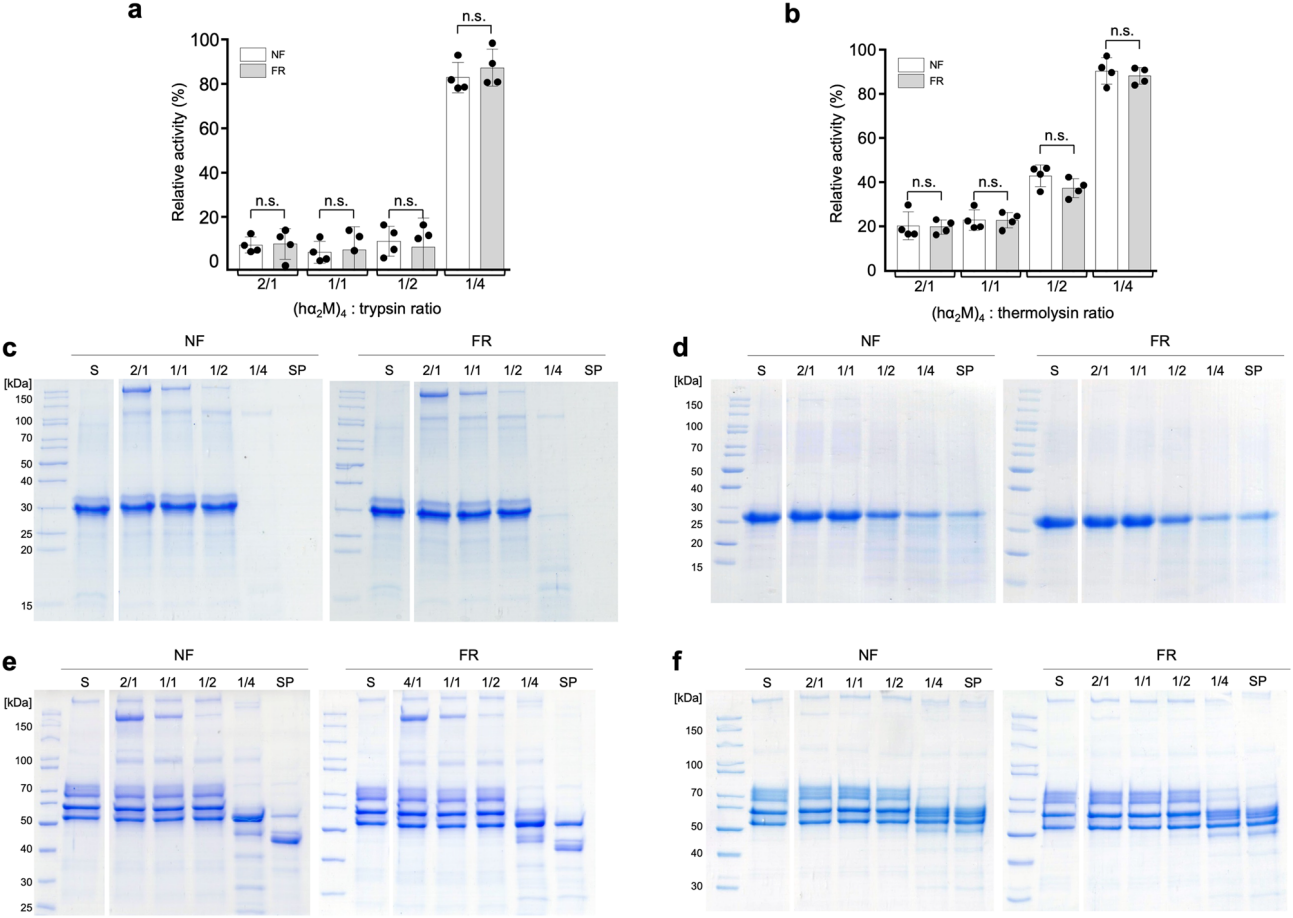
Inhibitory activity in vitro of hα_2_M from non-frozen fresh plasma (NF) or frozen fresh plasma (FR). Inhibition of (**a**) trypsin and (**b**) thermolysin activity against the fluorescent BODIPY FL-casein substrate. Percentages of remaining activity are represented as means ± SD (n = 3). Statistical significance was determined using a two-sided Student’s t-test (n.s., *p* > 0,1). (**c**–**f**) SDS-PAGE inhibition assay at different tetrameric hα_2_M:peptidase ratios but constant peptidase concentrations, showing the inhibition of trypsin activity against (c) α-casein and (e) fibrinogen, and the inhibition of thermolysin activity against (d) α-casein and (f) fibrinogen, for which only the Aα chain is cleaved. Lanes S and SP correspond to the substrate alone and the substrate incubated with and processed by the peptidase, respectively.

We compared the stoichiometry of inhibition in the FR and NF protein preparations by titrating hα_2_M against two model endopeptidases from different catalytic classes at various molar ratios. We evaluated the serine peptidase trypsin from bovine pancreas and the metallopeptidase thermolysin from *Bacillus thermoproteolyticus* by testing their activity against a fluorogenic casein derivative (Fig. 2a,b). Both endopeptidases showed equivalent residual activities after preincubation with native hα_2_M from the two preparations at inhibitor tetramer:peptidase molar ratios of 2:1, 1:1 and 1:2. This reflected the ability of cleaved substrate fragments, still labelled with the fluorophore, to access the trapped peptidase, leading to the emission of fluorescent signal as previously reported^54^. At the highest molar ratio (1:4), the inhibitor was saturated and the excess peptidase therefore led to a sudden increase of proteolytic activity (Fig. 2a,b). Again, the FR and NF preparations were indistinguishable, as in the preceding experiments.

Finally, we analysed the ability of hα_2_M to inhibit trypsin and thermolysin cleavage of α-casein (32 kDa) and fibrinogen (340 kDa, divided into the three chains Aα, Bβ and γ), which are good substrates for both peptidases, by SDS-PAGE (Fig. 2c–f). We found that hα_2_M inhibited both peptidases at molar ratios between 1:1 and 1:2, and the FR and NF samples were once again indistinguishable. Overall, these results indicate that freezing plasma does not affect the inhibitory activity of hα_2_M, suggesting it remains in its functional native conformation.

### Repeated freezing and thawing cycles of purified hα2M induce sample heterogeneity

**Figure 3:**
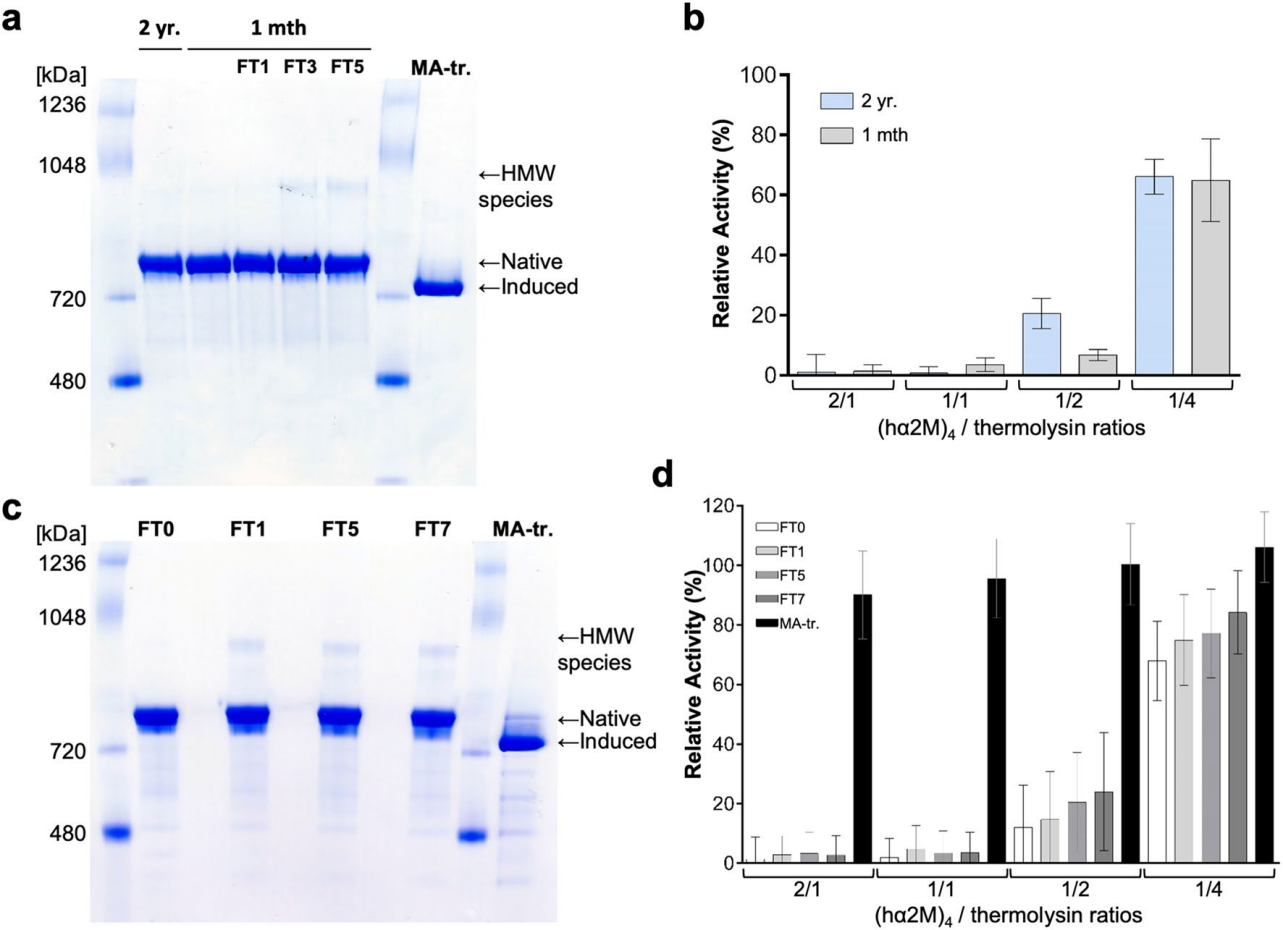
Effect of long-term storage and freezing/thawing of native hα_2_M. (**a**,** c**) Native PAGE analysis of native hα_2_M from FR samples stored for (a) 1 month or 2 years or (**c**) 3 months at – 30 °C. Native hα_2_M samples were further subjected to seven fast (**a**) or slow (**b**) freeze–thaw cycles (FTx). This procedure causes the emergence of high-molecular-weight (HMW) species, as previously reported for protein stored for several months at 4 °C^39^. MA-treated hα_2_M was used as a control for induced hα_2_M. (**b**,** d**) Inhibition of thermolysin activity against the fluorescent substrate BODIPY FL-casein by (**b**) hα_2_M from FR samples stored for 1 month or 2 years at – 30 °C and (**d**) hα_2_M samples subjected to freeze–thaw cycles. The percentages of remaining activity are represented as means ± SD (**b**: n = 3; **d**: n = 6). MA-treated hα_2_M is representative of the inactive inhibitor.

It has been reported that, once purified, native hα_2_M is more susceptible to freeze and thaw^39^. To assess this in the complex context of plasma, we analysed the biophysical and functional properties of pure native hα_2_M after several fast (Fig. 3a) or slow (Fig. 3c,d) freeze-and-thaw cycles (FTs). In agreement with^39^, fast freezing in liquid nitrogen in up to three FTs apparently did not significantly impair the protein and high-molecular-weight species, which are indicative of conformational changes as shown in Fig. 1 of^39^, appeared only after the third cycle (Fig. 3a). Contrary to^39^, however, we did not detect induced species, which we attribute to the presence of sodium azide in these published studies. This reagent is a chemical preservative and potent nucleophile^55^, which can attack the thioester bond and induce rearrangement in a similar fashion to MA. In contrast, slow FTs already caused biophysical heterogeneity after the first cycle (Fig. 3c). This correlated with an associated slight loss of inhibitory capacity against thermolysin (Fig. 3d).

Finally, we tested whether long-term storage at -30ºC impaired native hα_2_M, as the source of several past studies had been outdated plasma, which corresponds to frozen fresh plasma after three years. We assessed the above biophysical and functional properties of hα_2_M purified from FR stored for 2 years at – 30 °C (Fig. 3a,b). No significant differences could be detected when compared with the results after one month storage.

## Conclusions

The structure and biochemistry of hα_2_M, an essential multimeric and multi-domain protein with many physiological functions and orthologs across animal species, has been investigated for decades. However, structural analysis has been hampered by the conformational variability of the native state^23,27,28^. In addition, early reports showed that native preparations featured serine peptidase activity^56^. This suggested the presence of induced, peptidase laden hα_2_M particles, which were also identified by cryo-EM, supporting clinical studies that detected peptidase-complexed hα_2_M in the plasma of healthy individuals^13^. These structural studies identified several reaction intermediates between the fully native and fully induced conformations in theoretically native purified hα_2_M from FR and NF plasma^23,27^, disputing claims that native hα_2_M purified from NF plasma is homogeneous^40^.

The conformational variability of native hα_2_M in our cryo-EM study, which was based on the use of FR plasma^23^, raised concerns about its functionality^40^. This reflected the reported fact that purified hα_2_M is sensitive to storage, lyophilisation and freezing^37–39^ due to a stress-induced conformational rearrangement that renders the proteins inactive or dysfunctional^57^. To address this issue, we compared the biophysical and functional properties of hα_2_M from FR and NF, which was freshly prepared for immediate use following purification as in our structural studies^23^, and was not stored, frozen or lyophilized. Moreover, treatment of hα_2_M from FR with trypsin had resulted in a homogeneously induced sample in cryo-EM^23^.

Our results unambiguously showed that the two protein preparations were identical in terms of biochemical, biophysical and functional properties. This indicates that blood plasma shields hα_2_M against stress caused by freeze-thawing. In plasma, hα_2_M is surrounded by other macromolecules at high concentrations of 60–80 mg/mL^58^. This environment has a cryoprotective effect based on macromolecular crowding, which enhances the native structure and stability of proteins^59^. Specifically, plasma from healthy donors has a glucose content of 0.7–1.4 g/L^60^, which is consistent with the protective effect of sucrose on purified hα_2_M^39^. Moreover, plasma contains large amounts of serum albumin (60% of the total protein content), which plays an important role in modulating osmotic pressure. Its bovine counterpart is widely used as an additive for the preservation of tissues, cells and proteins such as lactate dehydrogenase^61^ and catalase^62^ during freezing.

The equivalence of our results with FR and NF plasma is also consistent with the fact that the plasma used in our studies over the last decade^23,26,31,50^ was obtained, fresh or frozen, from the only authorized blood bank in Catalonia and was not outdated. It conforms to the quality criteria of therapeutic fresh frozen plasma^48^, which is administered immediately after thawing to treat coagulation disorders^63^. The relevance of this reagent is highlighted by its inclusion on the WHO list of essential medicines^64^. Moreover, early clinical studies reported that fresh frozen plasma compensates for the loss of hα_2_M that occurs during early stages of pancreatitis^65^. These data together confirm that hα_2_M from FR plasma is functionally competent.

In summary, the storage of native hα_2_M in FR plasma before purification does not alter its properties. Indeed, FR plasma has been used in several laboratories worldwide for decades to prepare native hα_2_M for biochemical and structural studies^23,26,31,32,50,66–71^. Often, the FR plasma was outdated, which we here also found to be functionally competent. FR plasma therefore appears suitable for native hα_2_M preparation, as previously discussed^38^, and can be envisaged as a source of hα_2_M on demand without needing to follow the time-consuming procedures from blood banks or to rely on donations each time the protein is required.

## Methods

### Preparation and purification of native and MA-treated hα2M

All experiments were performed according to the applying guidelines and regulations and were approved by the Committee for Ethics in Research with Medicines and the Commission of Research Projects of the Vall d’Hebron University Hospital (Barcelona). NF plasma was obtained < 24 h after collection from anonymous healthy human donors (LST-BIOBANC, Banc de Sang i Teixits, National Government of Catalonia), so that no consent from the subjects was required. Each sample was anonymized and checked to be free of hepatitis viruses (HVB and HVC), *Treponema pallidum* ssp. *pallidum* (the syphilis pathogen) and human immunodeficiency virus (HIV). This plasma is suitable for transfusion into patients, is routinely stored at − 30 °C in the blood bank, and has a shelf-life of 3 years, thus conforming to the clinical standards of therapeutic fresh frozen plasma. Half of the sample was used immediately for purification without freezing (NF plasma) and the rest was frozen at − 20 °C within 1 h, stored at this temperature for 16 h and then used for purification (FR plasma) following the procedure of previous cryo-EM studies^23^. Subsequent sequential precipitation steps with 4–12% PEG 4000 were carried out at 4 °C as previously described^50^. The final precipitate containing hα_2_M was reconstituted in 20 mM sodium phosphate buffer (pH 6.8) supplemented with 5 mM phenylmethylsulfonyl fluoride. All subsequent purification steps were performed at 4 °C. The hα_2_M was first captured on a zinc-chelating resin (G-Biosciences) in an open column (Bio-Rad) and was then washed with 50 mM sodium phosphate buffer (pH 7.2) containing 250 mM sodium chloride and 10 mM imidazole before elution in 50 mM sodium phosphate buffer (pH 7.2) containing 250 mM sodium chloride and 100 mM ethylenediaminetetraacetate (EDTA). The eluate was exchanged to buffer A (20 mM sodium phosphate, pH 7.4) on a PD-10 column (Cytiva) followed by IEC on a TSKgel DEAE-2SW column (TOSOH Bioscience) equilibrated with buffer A. Fractions were eluted in 30 mL buffer B (20 mM sodium phosphate, 1 M sodium chloride, pH 7.4) applied as a 2–50% gradient vs. buffer A, and the central peak fractions were pooled and concentrated. Final purification was carried out by SEC on a Superose 6 10/300 column (GE Healthcare Life Sciences) in buffer C (20 mM Tris–HCl, 150 mM sodium chloride, pH 7.4). MA-treated hα_2_M (MA-hα_2_M) was obtained by reacting native hα_2_M, buffer-exchanged to 100 mM Tris–HCl (pH 8) after the IEC purification step, with 200 mM methylamine hydrochloride overnight at 4 °C, followed by SEC as described for the native counterpart.

### Sample freezing and thawing

Purified native hα_2_M was subjacted to fast or slow freeze–thaw (FT) cycles. Fast FT was performed by freezing in liquid nitrogen and thawing in a water bath at room temperature, while slow FT was performed by directly placing the samples at – 20 °C (freezing) and on ice (thawing).

### Proteolytic inhibition assays

Purified native hα_2_M was used to study the inhibition of bovine pancreatic trypsin and *Bacillus thermoproteolyticus* thermolysin (both from Sigma-Aldrich) after incubation for 10 min at room temperature. Reactions were carried out in buffer C using tetrameric hα_2_M:peptidase ratios of 2:1, 1:1, 1:2 and 1:4. Reaction products were used directly to monitor residual peptidase activity against fluorogenic and natural substrates at 37 °C. For the fluorogenic substrates, the activity of 50 nM peptidase was tested in a reaction volume of 100 μL in a Synergy H1 microplate fluorimeter (Biotek) using the EnzCheck Assay Kit, which includes 5 μg/mL BODIPY FL-casein (λ_ex_ = 505 nm and λ_em_ = 513 nm; Invitrogen). As natural substrates, we used α-casein from bovine milk (35 kDa) and fibrinogen from human plasma (340 kDa) at a concentration of 0.5 mg/mL (both from Sigma-Aldrich) with trypsin and thermolysin at 100 nM and 5 nM, respectively. Reactions were monitored for 10 min (thermolysin) or 90 min (trypsin), and cleavage was assessed by 10–14% Tricine-SDS-PAGE after stopping the reactions with small-molecule inhibitors (0.7 mM Pefabloc SC from Roche Life Sciences for trypsin and 20 mM EDTA for thermolysin) and subsequent heating for 5 min at 95 °C.

### SEC–MALLS

Samples were assessed in a Dawn Helios II device (Wyatt Technologies) coupled to a Superose 6 10/300 Increase SEC column (Cytiva) at the joint IBMB/IRB Crystallography Platform, Barcelona Science Park (Catalonia). The column was equilibrated in buffer C at 25 °C to analyse native and induced hα_2_M from FR and NF plasma. Data were processed and analysed using *ASTRA 7* software (Wyatt Technologies) and a *dn/dc* value typical for proteins (0.185 mL/g).

### Determination of free sulfhydryl groups

Preparations of native hα_2_M (after IEC) and MA-hα_2_M were exchanged to buffer C at a final concentration of 25 mg/mL, and free sulfhydryl groups were determined by reaction with Ellman’s reagent (5,5′-dithiobis-2-nitrobenzoic acid; Sigma)^51^ for 15 min. The change in A_412_ was monitored using a Power-Wave XS microplate spectrophotometer (Biotek). The concentration of free-thiol groups was calculated based on the molar extinction coefficient of Ellman’s reagent (14,150 M^−1^ cm^−1^;^72^), as previously done for hα_2_M samples by others^71^. The absorbance signal was measured in 96-well plates containing 200 μL of Ellman’s assay samples plus 20 μL of cysteine or test samples in triplicate.

### Miscellaneous

Protein identity and purity were assessed by 10–14% Tris–Glycine SDS-PAGE stained with Coomassie-brilliant blue using Unstained Protein Molecular Weight Marker (10–200 kDa; Thermo Fisher Scientific) and BlueStar Plus Prestained Protein Marker (10–240 kDa; NIPPON Genetics) as molecular-mass markers. Native protein samples were also analysed by native NuPAGE 3–8% Tris–Acetate Mini Protein Gels (Invitrogen) stained with Coomassie-brilliant blue, and NativeMark Unstained Protein Standard (20-to-1200 kDa; Invitrogen) was used as molecular-mass marker.

Ultrafiltration steps were performed with Vivaspin 15, Vivaspin 2 and Vivaspin 500 filter devices with cut-off values ranging from 50 to 100 kDa (Sartorius Stedim Biotech). Protein concentrations were estimated by measuring A_280_ values in a NanoDrop spectrophotometer and applying the corresponding theoretical extinction coefficients. Concentrations were also measured using the BCA Protein Assay Kit (Thermo Fisher Scientific) with bovine serum albumin as a standard.

## Supplementary Information

Supplementary Information.

## Data Availability

All data and reagents are freely available from Dr. Gomis-Rüth upon reasonable request.
