# Supplementary figures and images for: Frozen fresh blood plasma preserves the functionality of native human α2-macroglobulin
Source: Sci Rep. 2023 Mar 20;13:4579. doi: 10.1038/s41598-023-31800-8 (PMC10027685; doi:10.1038/s41598-023-31800-8)

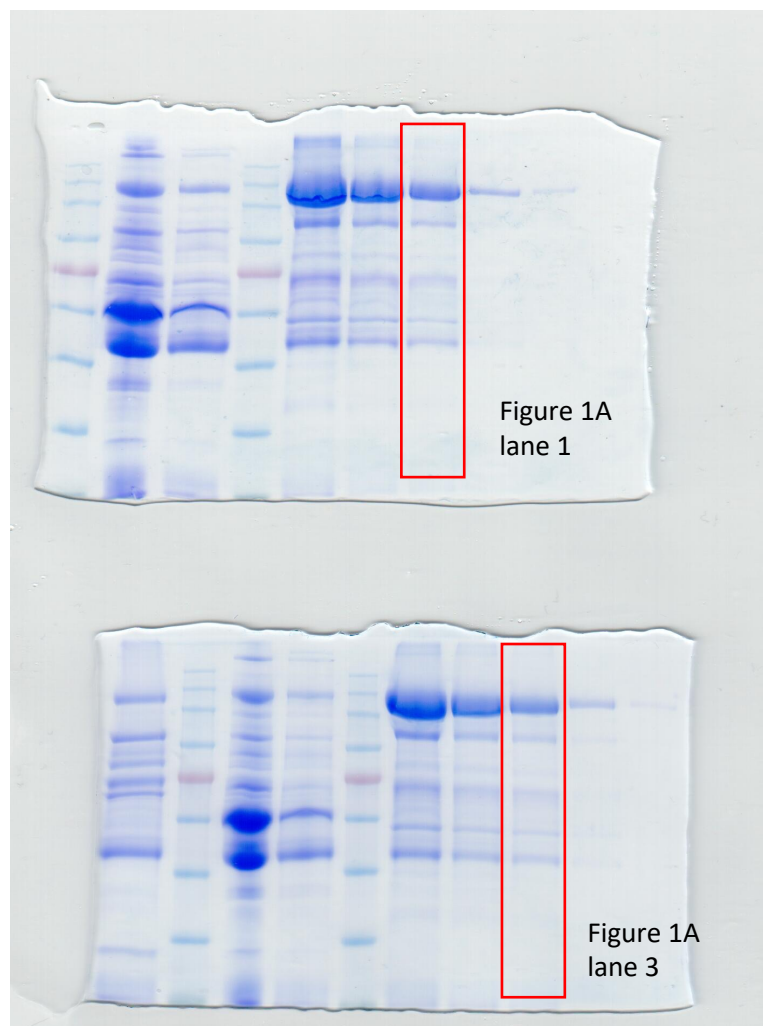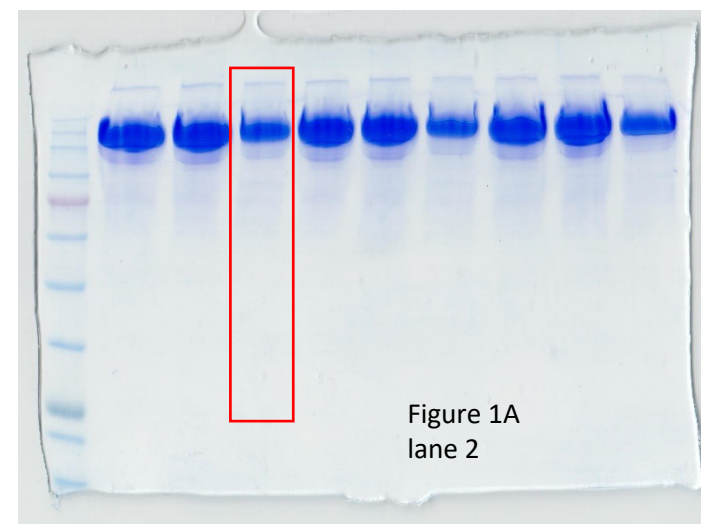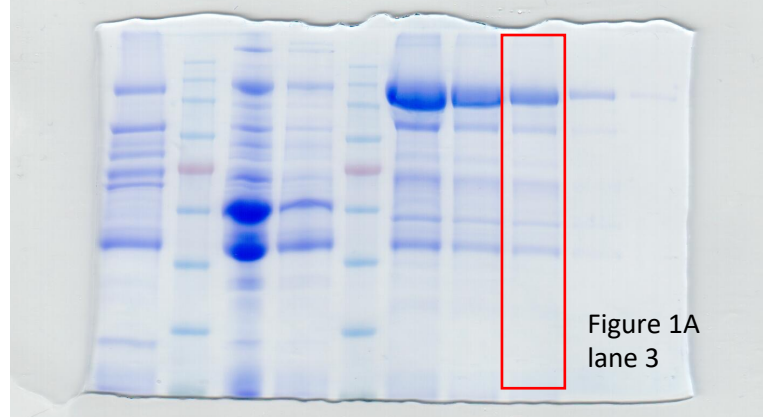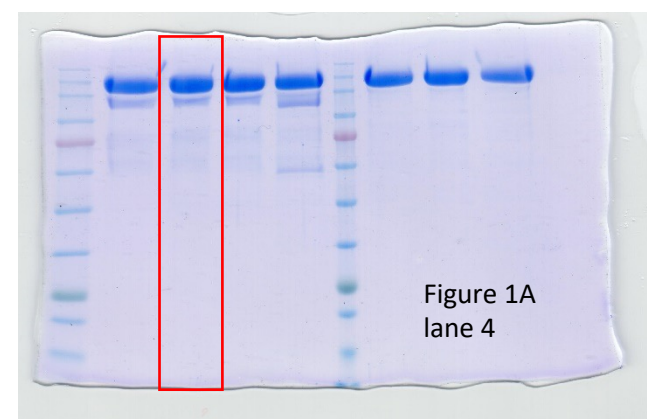

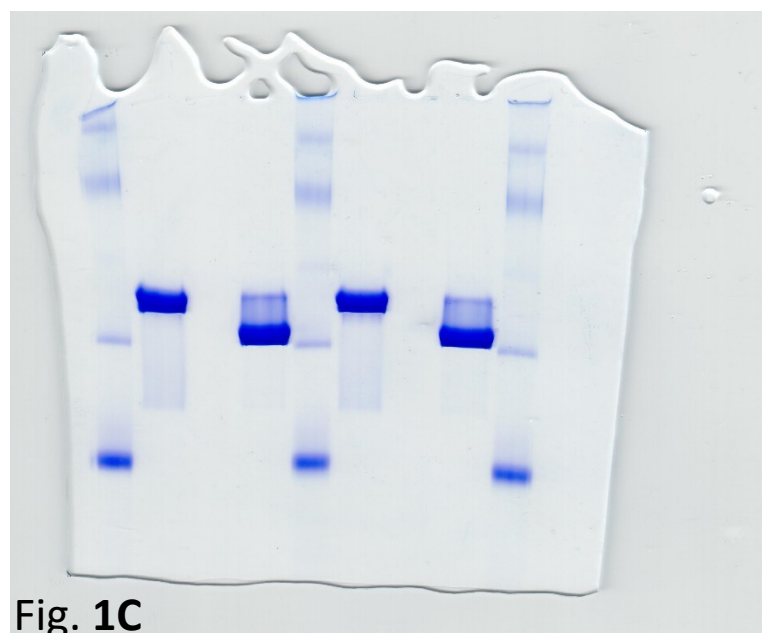

Fig. 1C

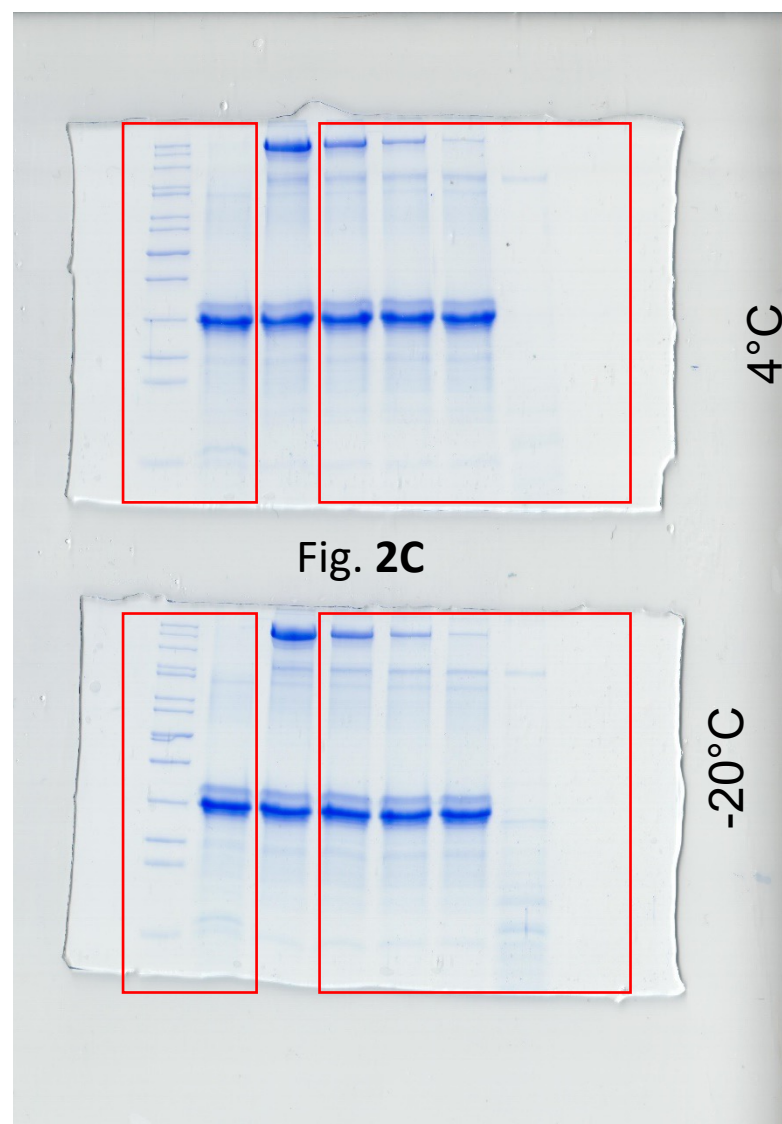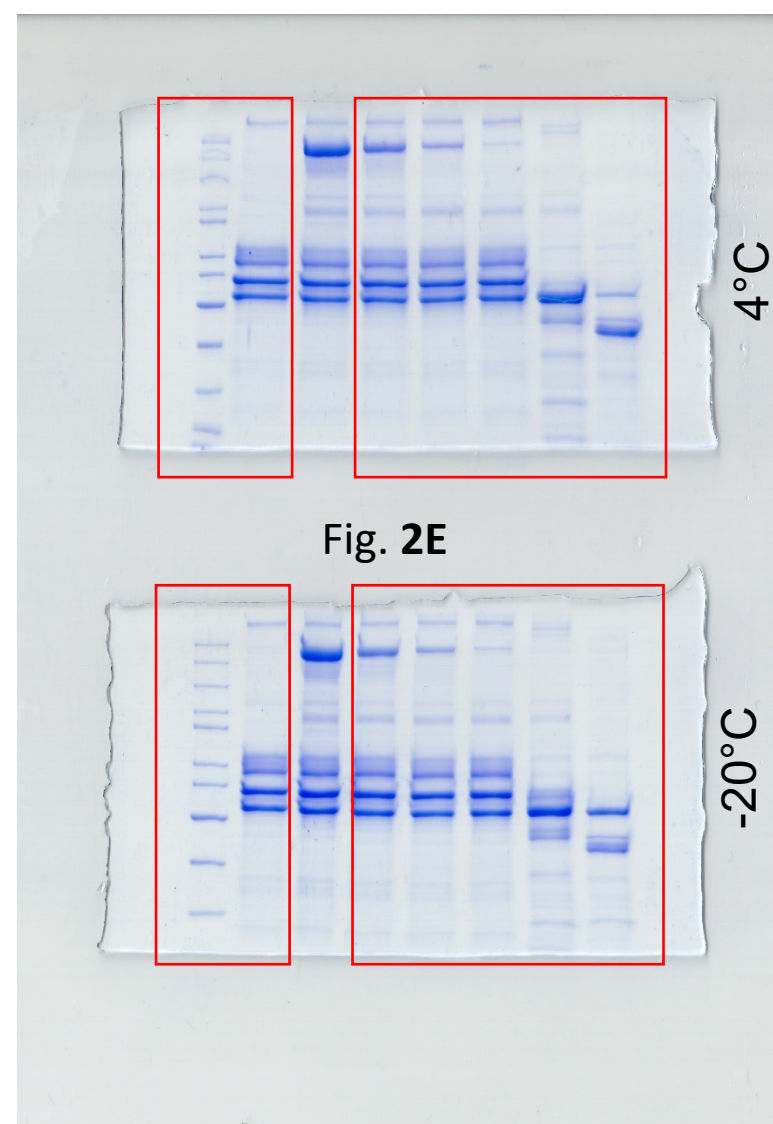

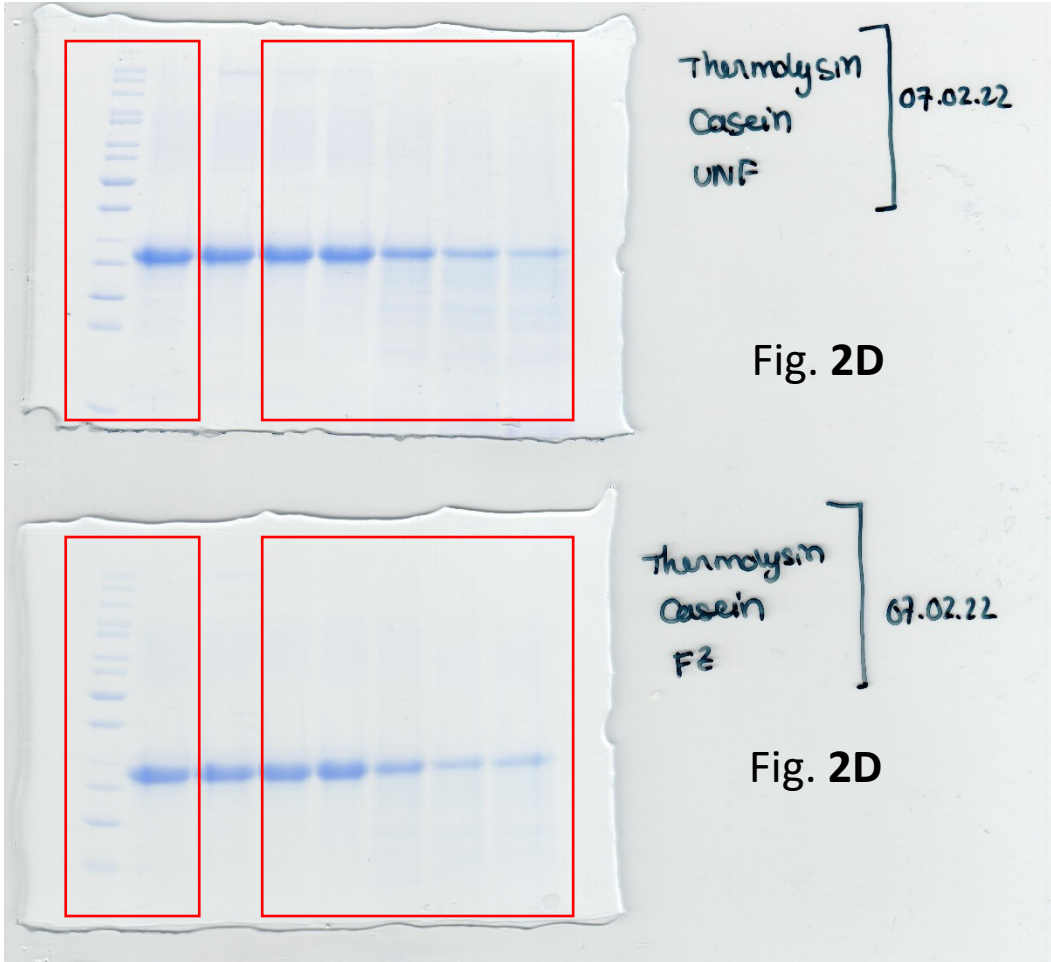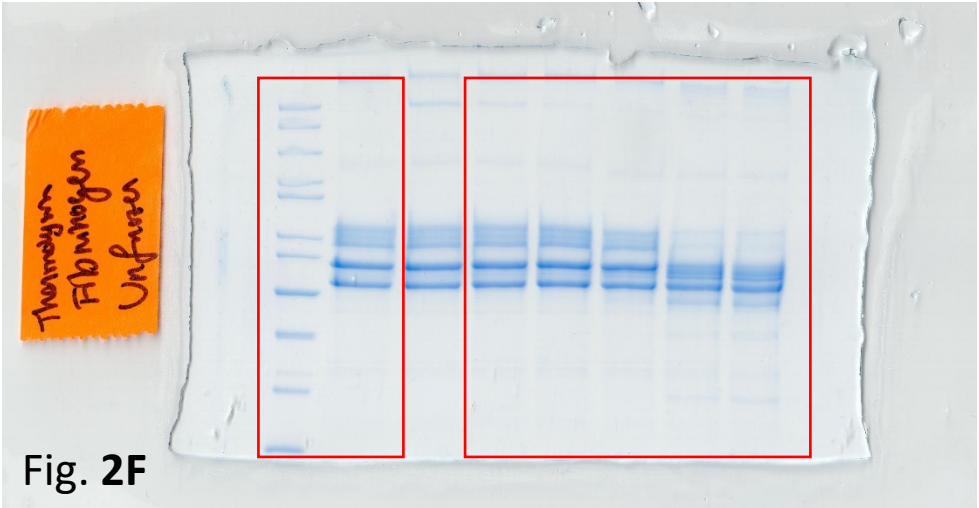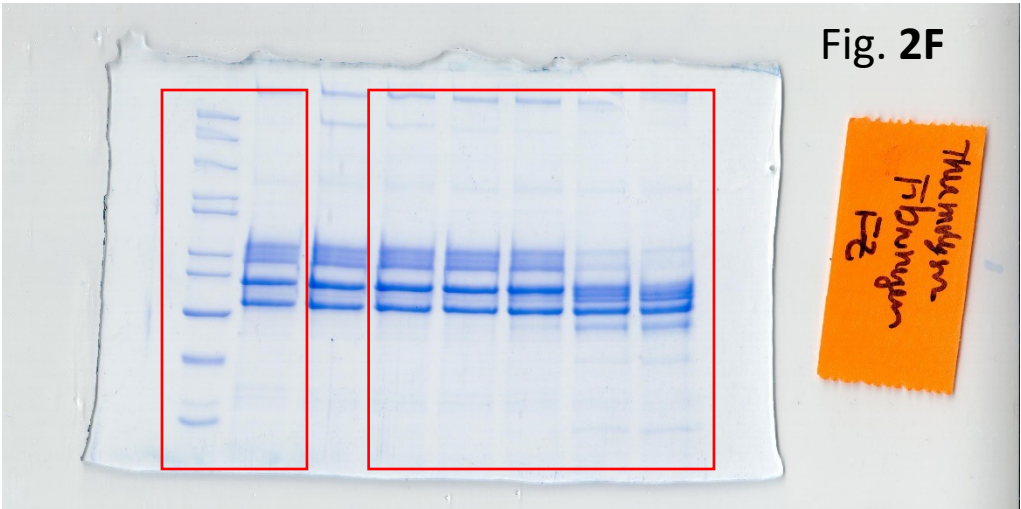

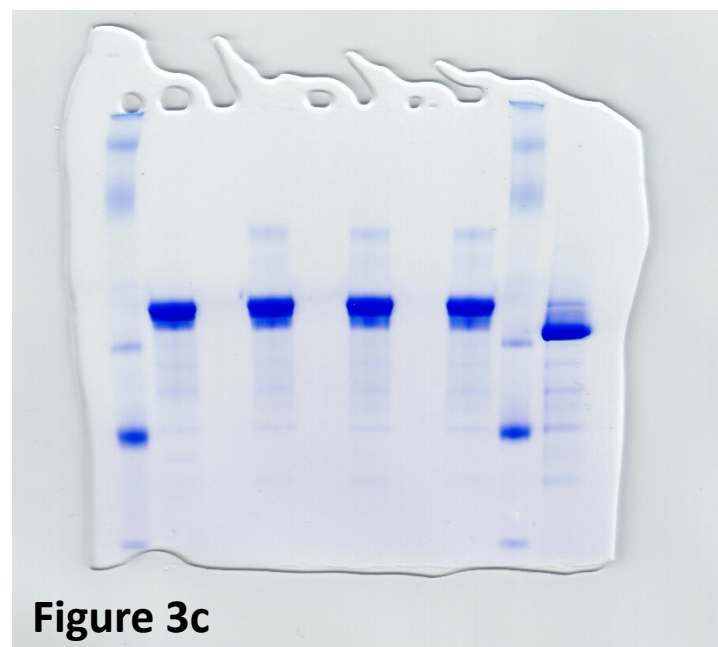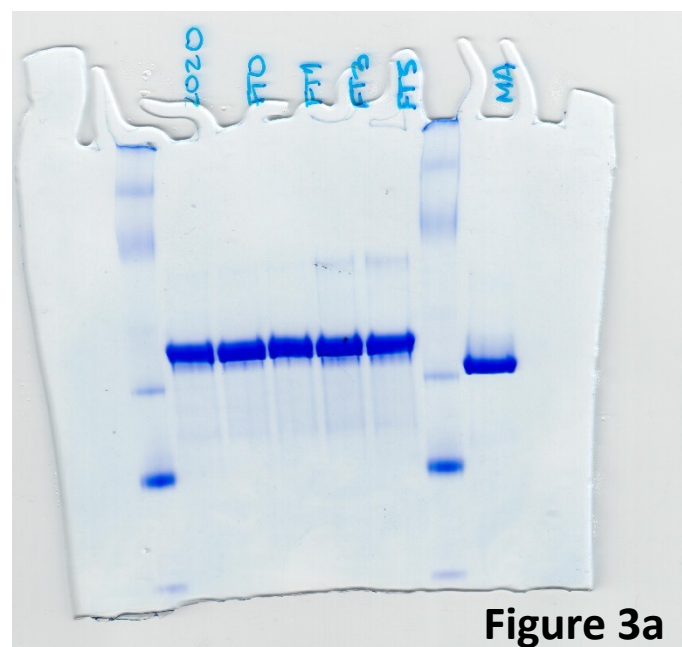

Supplement: Supplementary file 1 — Supplementary Information. [file 41598_2023_31800_MOESM1_ESM.pdf]
